# The impact of a freestyle rope-skipping intervention on resilience in junior high school students: the mediating role of self-efficacy

**DOI:** 10.3389/fpsyg.2026.1834465

**Published:** 2026-04-30

**Authors:** Wenqi Wan, Jiulong Song, Guoguo Zhao, Jiexin Gu, Jian Fu

**Affiliations:** College of Physical Education, Yangzhou University, Yangzhou, Jiangsu, China

**Keywords:** freestyle rope skipping, junior high school students, mediation, resilience, self-efficacy

## Abstract

**Objectives:**

This study examined whether an 8-week freestyle rope-skipping intervention improves resilience and self-efficacy among junior high school students, and whether self-efficacy serves as a partial mediator in the association between the intervention and resilience.

**Methods:**

Two eighth-grade classes (*N* = 90; aged 13–14 years) were assigned to either an experimental group (freestyle rope skipping; 30 min per session, three sessions per week for 8 weeks; *n* = 43) or a control group (regular running during the afternoon break; *n* = 47). Resilience was assessed using the Chinese version of the Connor–Davidson Resilience Scale (CD-RISC), and self-efficacy was measured using the General Self-Efficacy Scale at both the pre-test and post-test. A 2 × 2 mixed model (Group Time) was used to test intervention effects, and PROCESS macro (Model 4) with 5,000 bootstrap resamples, while statistically accounting for baseline levels of the intervening variable and outcome included as covariates.

**Results:**

At post-test, students in the rope-skipping class scored significantly higher of resilience (102.58 ± 12.54 vs. 77.51 ± 14.86) and self-efficacy (34.63 ± 4.37 vs. 26.66 ± 9.37) compared to the control group (both *p* < 0.01). A significant positive correlation emerged between resilience and self-efficacy scores (r = 0.508, *p* < 0.01). Path analysis revealed that self-efficacy partially accounted for the association between the intervention and resilience (indirect effect = 0.229, 95% bootstrap CI [0.063, 0.394]; direct effect = 1.118; total effect = 1.346).

**Conclusion:**

An 8-week freestyle rope-skipping intervention was associated with significant improvements in resilience and self-efficacy among junior high school students compared with regular running. Moreover, self-efficacy served as a partial mediator in the association between the intervention and resilience, suggesting that improvements in self-efficacy may represent one pathway through which school-based physical activity supports resilience.

## Graphical abstract

**Figure:**
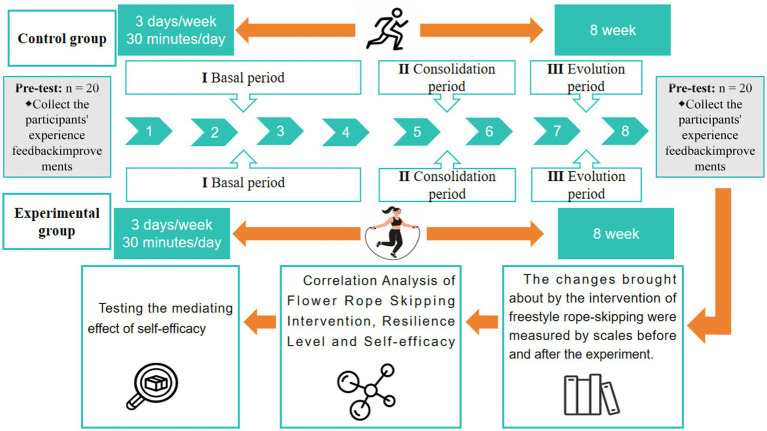

## 1 Introduction

The rising incidence of mental health difficulties among adolescent populations has increasingly been recognized as a pressing public health issue worldwide. The World Health Organization reports that approximately one in seven adolescents worldwide experiences a mental health disorder (Kieling et al., 2024). In China, epidemiological data indicate a prevalence rate of 17.5% for mental disorders among children and adolescents aged 6–16, suggesting that nearly 38 million young people may suffer from psychological difficulties such as anxiety or depression (Xia et al., 2019). As a key developmental stage, junior high school students encounter substantial academic, social, and emotional challenges, making their psychological well-being a priority for research and intervention.

Within the field of adolescent mental health, resilience is frequently conceptualized as the ability to maintain positive adaptation when encountering adverse circumstances (Bonanno, 2004; Epstein and Krasner, 2013; Panter-Brick and Leckman, 2013; Anderson and Priebe, 2021). During early adolescence, students face increasing academic demands, peer competition, and developmental stressors, which place greater demands on their resilience (Steare et al., 2023). However, rising academic pressure (Fenwick-Smith et al., 2018), intensified peer competition(Nobre et al., 2021), and the pervasive use of electronic devices (Santos et al., 2023) have collectively reduced adolescents’ engagement in physical activity, thereby exacerbating risks to their physical and mental health. A growing body of evidence suggests that physical exercise plays a crucial role in promoting mental health and psychological resilience, partly through psychological mechanisms that enhance individuals’ coping resources and adaptive capacities (Yubin et al., 2025). One psychological factor closely related to resilience is self-efficacy, defined as an individual’s belief in their capacity to organize and execute the actions necessary to manage prospective situations (Bandura, 2004). Higher self-efficacy enables individuals to approach challenges with confidence, persist in the face of difficulties, and employ more adaptive coping strategies—key characteristics of resilient functioning (Kaniasty et al., 2025). During early adolescence, self-efficacy is vital for emotional regulation, motivation, and sustained engagement in adaptive behaviors, thereby contributing to adolescents’ ability to cope with stress and recover from adversity (Serey et al., 2019). Research in sport and exercise psychology has consistently shown a reciprocal relationship between physical activity and self-efficacy: participation in physical exercise enhances self-efficacy through mastery experiences and social interaction, and this increased self-efficacy, in turn, facilitates adaptive coping and persistence when facing challenges, thereby contributing to the development of psychological resilience (Wang et al., 2022; Zhu et al., 2023).

This investigation is grounded in Bandura’s Social Cognitive Theory (SCT), which serves as the overarching theoretical lens, offering a valuable perspective on how physical activity may influence adolescents’ psychological resilience. SCT posits that human functioning results from the dynamic interplay among personal factors, behavior, and the environment, with self-efficacy serving as a central cognitive mechanism that regulates behavioral effort, persistence, and emotional responses (Bandura, 1997). Therefore, according to SCT, self-efficacy may function as a key psychological pathway linking physical activity to the development of resilience in adolescents (Jiang et al., 2025; Peng et al., 2025). While regular physical activity enhances cardiovascular, neurological, and musculoskeletal functioning and provides repeated exposure to manageable stressors that facilitate emotional regulation (Marconcin et al., 2022), different forms of activity may exert distinct psychological effects. Compared to monotonous aerobic exercises like continuous running, activities that incorporate progressive skill acquisition, coordination demands, and immediate performance feedback may be more effective in enhancing perceived competence and self-efficacy (Rodgers et al., 2014; Costigan et al., 2016).

Resilience, defined as the capacity for positive adaptation in the face of adversity, is theoretically intertwined with self-efficacy (Sagone et al., 2020). Individuals with stronger efficacy beliefs are more likely to adopt problem-focused coping strategies, persist through challenges, and regulate negative emotions effectively. It is hypothesized that an 8-week freestyle rope-skipping intervention may enhance self-efficacy through rich mastery experiences and social interaction, which in turn partially contributes to improvements in resilience.

Freestyle rope skipping was selected as the intervention for several reasons. First, its progressive skill acquisition—from basic jumps to complex routines—provides repeated mastery experiences, a key source of self-efficacy (Lee and Evans, 2019; Maria et al., 2025). Second, it integrates individual practice with group coordination (e.g., paired skipping, long-rope routines), offering social interaction and peer modeling that may strengthen efficacy beliefs (Pujari, 2024). Third, its progressive difficulty structure exposes students to manageable challenges while providing rapid skill feedback and high enjoyment, which promote sustained engagement and coping skill development (Ruby et al., 2011; Li et al., 2024). These characteristics make freestyle rope skipping particularly suitable for examining how structured physical activity may influence self-efficacy and, in turn, resilience in adolescent populations (Deuster and Silverman, 2013; Huang et al., 2024; Xia et al., 2024).

Despite growing interest in rope-skipping-based interventions, empirical evidence regarding their effects on resilience among junior high school students remains limited. In particular, few experimental studies have systematically examined the underlying psychological mechanisms, particularly whether self-efficacy serves as an explanatory factor. Clarifying such mechanisms is essential for optimizing intervention design and informing school-based mental health promotion strategies. Therefore, the present study aimed to evaluate the effects of an 8-week freestyle rope-skipping intervention on resilience and self-efficacy among junior high school students and to examine whether self-efficacy mediates the relationship between the intervention and resilience.

### 1.1 Hypotheses

*H1:* The freestyle rope-skipping intervention will significantly improve resilience among junior high school students.

*H2:* The intervention will significantly enhance students’ self-efficacy.

*H3:* Self-efficacy will function as a partial mediator in the intervention-resilience association.

## 2 Method and materials

### 2.1 Study design

This study employed a quasi-experimental design. Two intact eighth-grade classes from a junior high school were selected using cluster sampling and were randomly assigned to either the experimental group or the control group. A 2 (Group: experimental vs. control) × 2 (Time: pre-test vs. post-test) mixed design was used to examine the effects of the intervention. The freestyle rope-skipping program served as the independent variable, resilience as the dependent variable, with self-efficacy specified as the intervening variable of interest.

### 2.2 Participants

An *a priori* power analysis was conducted using G*Power 3.1. Assuming a medium effect size (d = 0.50), *α* = 0.05, and power = 0.80, the required minimum sample size was 36 participants per group. The final sample (*N* = 90) exceeded this requirement. Using cluster sampling, two intact eighth-grade classes were recruited and randomly assigned to the rope-skipping class (*n* = 45) or the running class (*n* = 45). All participants were aged 13–14 years. The inclusion criteria were as follows: (1) good physical health with no movement disorders; (2) absence of genetic or congenital diseases; (3) absence of cardiovascular diseases; and (4) no participation in regular extracurricular physical exercise during the intervention period. Participants who did not meet these criteria or who failed to comply with the study protocol were excluded from the final analysis.

Prior to participation, written informed consent was obtained from all students and their parents or legal guardians. The study was approved by the Ethics Committee of Yangzhou University (Approval No. YXYLL-2024-127).

### 2.3 Measurement instruments

We used the Chinese version of the Connor-Davidson Resilience Scale (CD-RISC) and the General Self-Efficacy Scale (GSES) to assess resilience and self-efficacy. Both scales have been culturally adapted and validated for use among Chinese students. In this study, Cronbach’s *α* was 0.87 for the CD-RISC and 0.88 for the GSES.

#### 2.3.1 Resilience

To measure resilience, participants completed the Chinese adaptation of the Connor-Davidson Resilience Scale (CD-RISC). This instrument was originally developed by Connor and Davidson (2003) and subsequently validated for use in Chinese contexts by Xiao and Zhang (2007) and Connor and Davidson (2003). The scale comprises 25 items, each rated on a 5-point Likert-type scale anchored by 1 (“never”) and 5 (“always”). Total scores are computed by summing item responses, with higher values reflecting more pronounced resilience.

#### 2.3.2 Self-efficacy

Self-efficacy was measured using the General Self-Efficacy Scale (GSES), which consists of 10 items rated on a 4-point Likert scale. The scale has been validated for use among Chinese primary and secondary school students and comprises two sub-dimensions: problem-solving (6 items) and coping/persistence (4 items) (34). Higher total scores reflect greater perceived self-efficacy. In the present study, Cronbach’s *α* was 0.882 for the full scale, indicating good internal consistency. All instruments have demonstrated acceptable reliability and validity in adolescent populations. Responses were collected in the classroom under the supervision of research assistants to ensure standardized administration.

### 2.4 Intervention procedures

#### 2.4.1 Control group

Participants in the control group engaged in routine running exercises during the afternoon break. The running sessions were conducted three times per week for 30 min per session over 8 weeks, matching the frequency, duration, and intervention period of the experimental group. All sessions took place on the school playground, where students ran collectively along designated routes at prescribed rhythms under the supervision of physical education teachers. Students were instructed to maintain orderly formations and follow standardized instructions throughout the exercise.

To minimize potential differences in exercise dose, both groups were exposed to comparable management intensity, including identical session duration, frequency, time of day, and supervision by physical education teachers. Exercise intensity in both groups was regulated according to standardized school physical education guidelines, with teachers providing real-time verbal instructions to maintain a moderate level of exertion. Both interventions were conducted in similar venues and under comparable organizational conditions to reduce attention-related and contextual effects.

It should be noted that the control group engaged in regular running, which may also confer psychological benefits (e.g., mood enhancement, stress reduction) and thus serves as an active control rather than a no-intervention control. This design was chosen to (a) control for the potential confounding effects of time, attention, and general physical activity exposure, and (b) evaluate the relative effectiveness of freestyle rope skipping compared to a typical school-based physical activity (i.e., running). However, this design does not allow for conclusions about the absolute effect of rope skipping relative to no physical activity. This limitation is addressed in the Discussion section.

#### 2.4.2 Freestyle rope-skipping intervention

The freestyle rope-skipping program was developed based on junior high school curriculum guidelines and the book *S*p*orts Games*. The intervention incorporated progressively challenging activities, including basic rope-skipping movements (e.g., side swings, parallel jumps, alternate-foot jumps, jumping jacks, and lunge jumps), double-person rope skipping, and group long-rope routines (e.g., 10-person “8-shaped” formations). The program integrated both short- and long-rope techniques, with at least five fundamental jump patterns practiced. Instruction was individualized according to each student’s skill level (see Supplementary Materials).

The program consisted of 24 sessions over 8 weeks (three sessions per week), each lasting 30 min of core skill-based training, preceded by warm-up (5–10 min) and followed by cool-down (5–10 min). Skill-based training progressed from basic single jumps to advanced freestyle routines, including cross-arm techniques, multiple rotations, paired routines, and coordinated group performances, culminating in a friendly in-class rope-skipping competition.

The training was designed for moderate-to-vigorous intensity (60–80% of estimated maximal heart rate, ~140–160 bpm). During each session, 20 randomly selected participants from both classes wore heart rate monitors (Polar H10), with data recorded at 5-min intervals to verify intensity. Participants were encouraged to maintain continuous activity during skill-based exercises, with brief rest periods provided when necessary. All sessions were conducted by experienced physical education teachers who had received prior training in freestyle rope-skipping techniques and the intervention protocol. To ensure consistency and standardization, instructors followed a structured lesson plan detailing session content, skill progression, feedback strategies, and safety procedures. Attendance was recorded at each session using a real-name sign-in system, and adherence to the intervention protocol was monitored by both instructors and research assistants to ensure active participation. Any missed sessions were documented, and participants were encouraged to make up missed training whenever feasible.

#### 2.4.3 Implementation and intensity control

Attendance was recorded at each session. Participants attending < 80% of sessions (i.e., missing ≥ 5) were excluded. Mean attendance was 94.3% (SD = 5.8) in the rope-skipping class and 92.7% (SD = 6.2) in the running class; no participants were excluded for low attendance.

To verify intensity, 20 randomly selected participants per session (from both classes) wore heart rate monitors. Data recorded at 5-min intervals confirmed that intensity remained within 140–160 bpm in both classes.

#### 2.4.4 Experimental control

Several measures were implemented to ensure experimental rigor:

1. Group homogeneity: Baseline measures of age, gender, resilience, and self-efficacy were collected to confirm equivalence between groups.
2. Intervention consistency: All sessions followed a standardized protocol conducted during afternoon recess to avoid interference with regular teaching.
3. Safety monitoring: Extracurricular physical activities were monitored throughout the intervention, and no adverse events were reported.

After screening, 10 participants were excluded due to incomplete participation, involvement in extracurricular sports training, or health-related issues. The final sample consisted of 90 participants.

### 2.5 Experimental procedure

**Figure 1:**
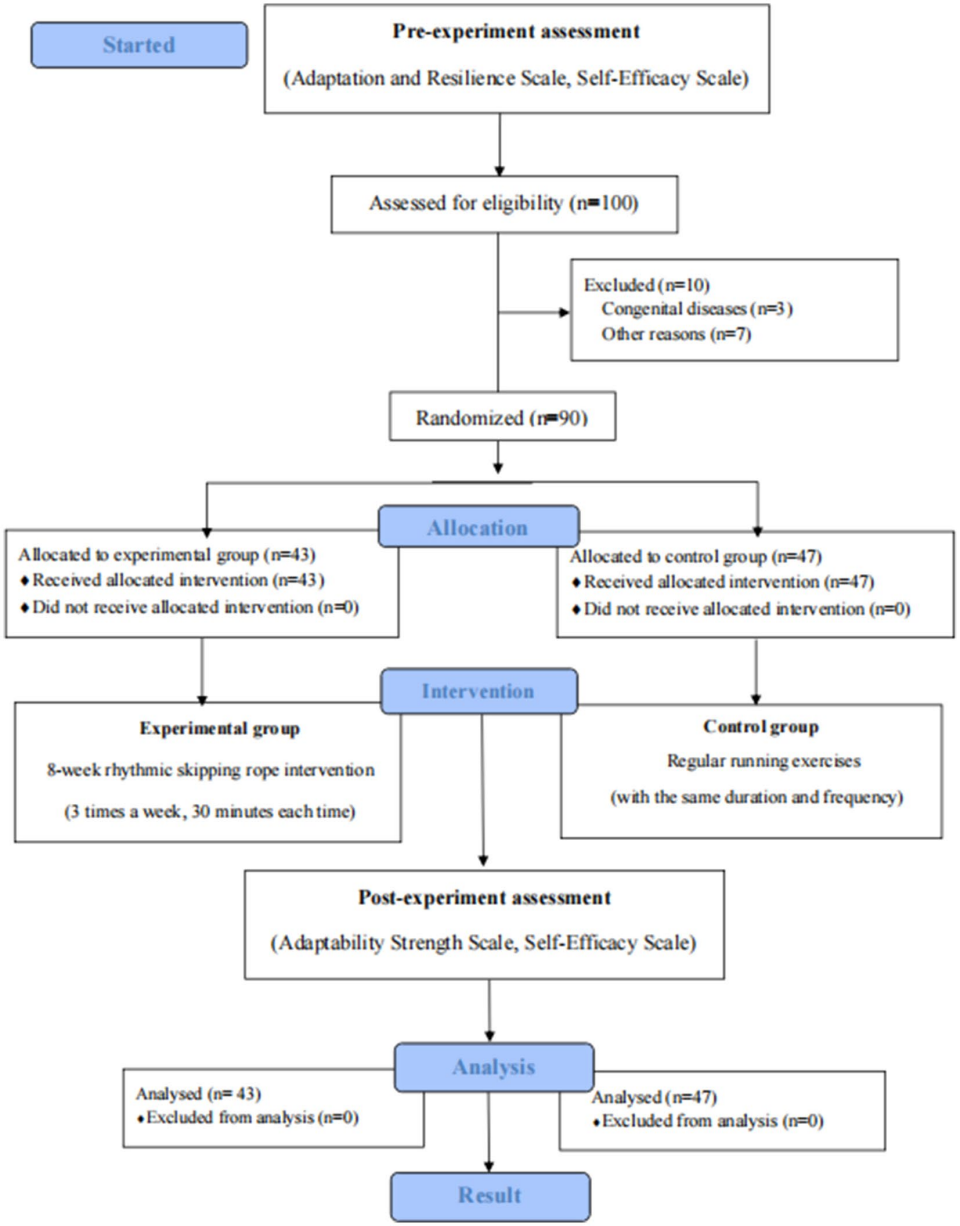
Experimental flowchart.

The study consisted of five stages: preparation, pilot testing, baseline assessment, intervention implementation, and post-intervention assessment. The research team received standardized training regarding intervention procedures, safety considerations, and data collection. A two-week pilot study involving 20 randomly selected students was conducted to assess feasibility and refine the intervention protocol. One week before the formal intervention, baseline assessments of resilience and self-efficacy were administered in classroom settings. After the 8-week intervention, the same instruments were administered again to assess the effects of the intervention. The overall experimental procedure is illustrated in Figure 1.

### 2.6 Statistical analysis

All analyses were conducted according to the per-protocol principle. Descriptive statistics were calculated for all variables. Normality and homogeneity of variance assumptions were assessed using the Shapiro–Wilk test, Q–Q plots, and Levene’s test. Primary intervention effects were examined using a Group × Time mixed-model ANOVA, with the interaction effect as the primary outcome of interest. Effect sizes and 95% confidence intervals are reported. To examine the indirect effect of the intervention on resilience through self-efficacy, a bootstrapped path analysis was performed using PROCESS macro (Model 4) for SPSS (Hayes, 2013) with 5,000 resamples. Baseline self-efficacy and resilience were included as covariates. Analyses were conducted at the individual level as the small number of clusters (two classes) precluded multilevel modeling. Statistical analyses were performed using SPSS version 27.0 (IBM Corp., Armonk, NY, USA). Statistical significance was set at **p < 0.05 (two-tailed), with Bonferroni correction applied for multiple comparisons.

Given that all variables were collected via self-report from the same participants at the same time points, we assessed potential common method bias using Harman’s single-factor test (Aguirre-Urreta and Hu, 2019). All items from the resilience and self-efficacy scales were entered into an unrotated exploratory factor analysis. The analysis revealed 12 factors with eigenvalues greater than 1. The first unrotated factor accounted for 19.635% of the total variance, which is below the recommended threshold of 40%, indicating that common method bias is unlikely to have substantially influenced the results.

## 3 Results

### 3.1 Participant characteristics

As shown in Table 1, no statistically significant differences were observed between the experimental and control groups in terms of age (13.36 ± 0.49 years vs. 13.53 ± 0.51 years, *p* = 0.094), sex distribution (male: 55% vs. 53%, *χ^2^* = 0.862, *p* = 0.926), total resilience score (78.30 ± 13.54 vs. 78.67 ± 11.34, *p* = 0.581), or total self-efficacy score (24.77 ± 5.39 vs. 24.53 ± 4.46, *p* = 0.450). These findings indicate that the baseline characteristics of the two groups were well balanced and comparable. As reported in the Statistical Analysis section, Harman’s single-factor test indicated that common method bias was unlikely to have substantially influenced the results.

**Table 1:** Baseline characteristics of participants (*n* = 90).

Variable	Control group (*n* = 47)	Experimental group (*n* = 43)	*χ^2^*	*F/t*	*p*-value
Age (years)	13.36 ± 0.49	13.53 ± 0.51	–	2.874	0.094
Sex, *n* (%)			0.862	–	0.926
Male	26 (55%)	23 (53%)			
Female	21 (45%)	20 (47%)			
Resilience total score	78.30 ± 13.54	78.67 ± 11.34		0.307	0.581
Self-efficacy total score	24.77 ± 5.39	24.53 ± 4.46		0.575	0.450

### 3.2 Primary outcomes

As presented in Table 2, repeated-measures ANOVA revealed significant Group × time interaction effects for both resilience and self-efficacy scores (*p* < 0.01). Simple effects analyses showed that resilience and self-efficacy scores increased significantly in the experimental group following the intervention compared with baseline levels (*p* < 0.01). In contrast, no significant changes were observed in the control group. In addition, post-intervention scores for both outcomes were significantly higher in the experimental group than in the control group (*p* < 0.01).

**Table 2:** The change of each indicator of subjects in two groups.

Characteristic	Control group (*n* = 47)	Experimental group (*n* = 43)	*p*-value
Resilience total score			
Pre-test	78.30 ± 13.54	78.67 ± 11.34	0.581
Post-test	77.51 ± 14.86**	102.58 ± 12.54**	**<0.01**
Self-efficacy total score			
Pre-test	24.77 ± 5.39	24.53 ± 4.46	0.450
Post-test	26.66 ± 9.37**	34.63 ± 4.37**	**<0.01**

Data are presented as mean ± standard deviation. **indicates a statistically significant difference between pre-test and post-test within the same group (*p* < 0.01). *p*-values in the last column represent between-group comparisons at the corresponding time point.

**Figure 2:**
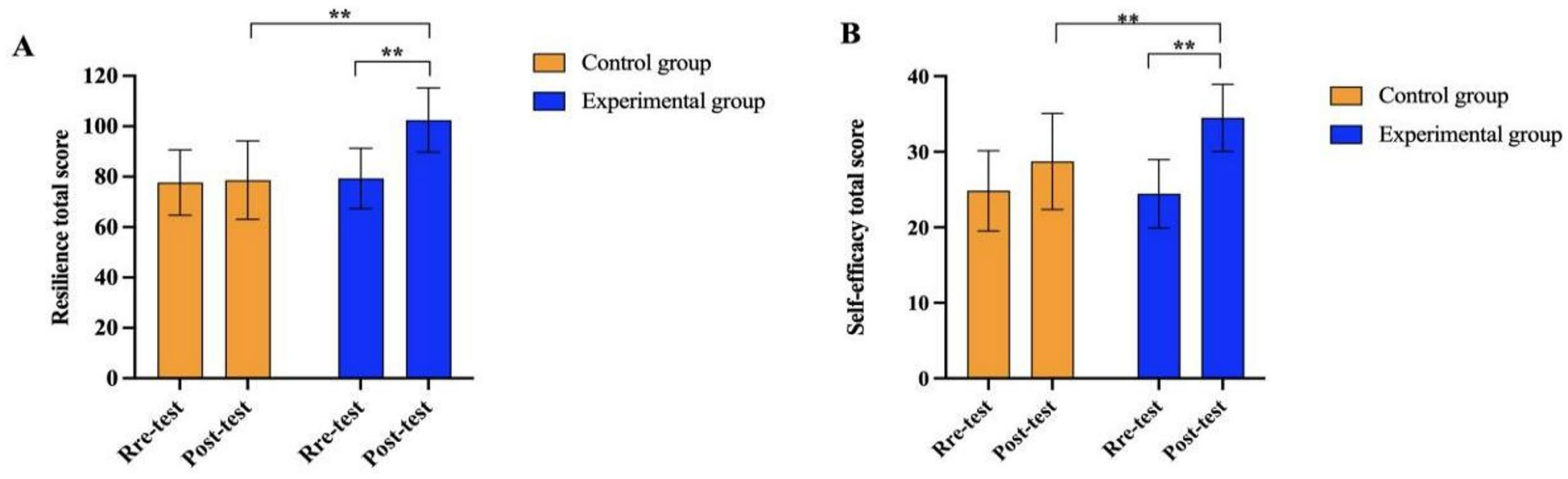
Changes in resilience and self-efficacy scores in the two groups. **(A)** shows the inter-group changes in the total score of psychological resilience, and **(B)** shows the inter-group changes in the total score of self-efficacy; “Pre-test” refers to the pre-intervention test, and “Post-test” refers to the post-intervention test; ** indicates that the between-group differences are statistically significant at *p* < 0.01.

As illustrated in Figure 2, both resilience (Figure 2A) and self-efficacy (Figure 2B) scores in the experimental group exhibited a clear upward trend following the intervention. In contrast, scores in the control group remained relatively stable across the two measurement points. This visual pattern is consistent with the statistical findings and further supports the presence of a significant interaction effect between Group and Time.

### 3.3 Correlation analysis

As shown in Table 3, statistically significant positive correlations were observed among participation in the rope-skipping intervention, resilience, and self-efficacy (*p* < 0.01). These results indicate that greater engagement in freestyle rope skipping was associated with higher levels of both resilience and self-efficacy among eighth-grade students.

**Table 3:** Correlation analysis results.

Variable	Freestyle rope skipping	Resilience	Self-efficacy
Freestyle rope skipping	1		
Resilience	0.676**	1	
Self-efficacy	0.477**	0.508**	1

Values represent Pearson correlation coefficients. ** indicates a statistically significant correlation (*p* < 0.01).

### 3.4 Mediation effect

**Figure 3:**
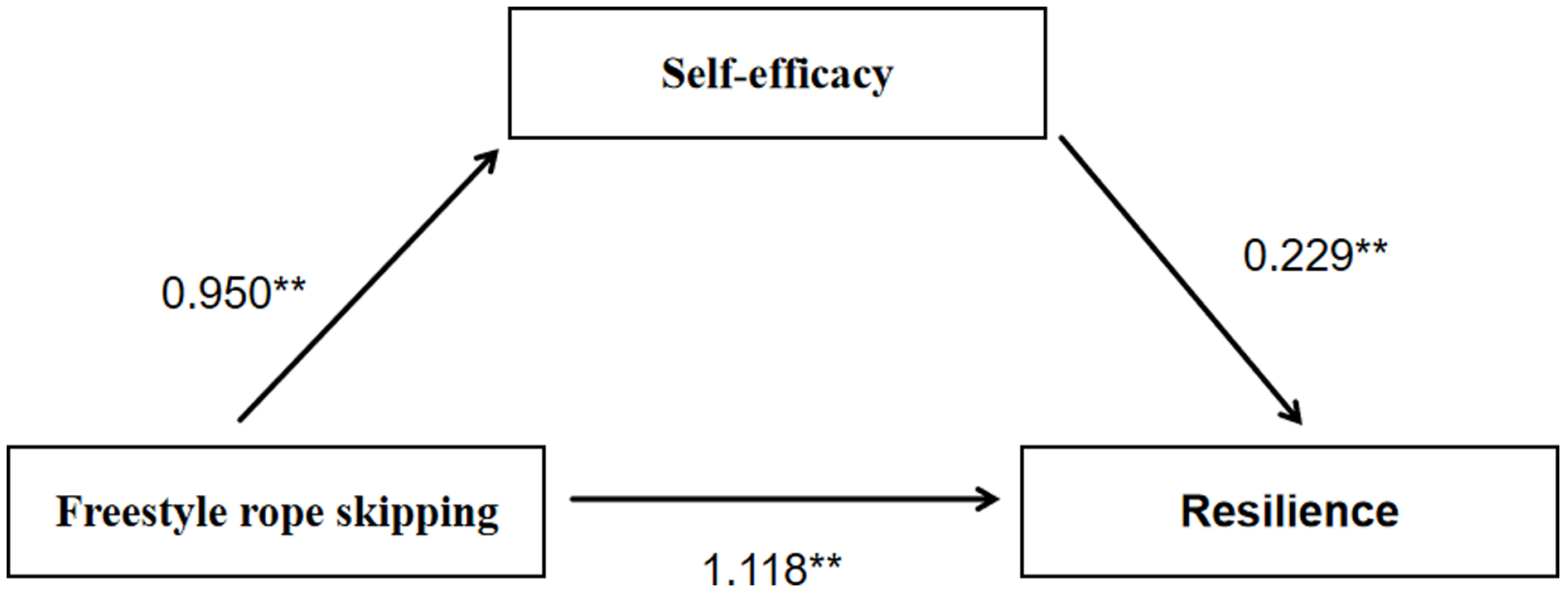
Model diagram of the mediating effect of self-efficacy between freestyle rope skipping and resilience. **indicates that the path coefficients are statistically significant at *p* < 0.01.

To determine whether self-efficacy functioned as a mediator in the association between the freestyle rope-skipping intervention and resilience, a mediation analysis was undertaken using the PROCESS macro (Model 4) with bootstrap resampling. As shown in Table 4, class assignment (rope-skipping vs. running) significantly predicted self-efficacy (*β* = 0.950, SE = 0.187, *t* = 5.091, *p* < 0.01), accounting for 22.8% of the variance in self-efficacy. When both the intervention and self-efficacy entered into the model, self-efficacy significantly predicted resilience, while the intervention’s direct effect on resilience retained statistical significance (*β* = 1.118, *SE* = 0.171, *t* = 6.520, *p* < 0.01). The model explained 50.2% of the variance in resilience. The mediation model is illustrated in Figure 3. Furthermore, the total effect of the intervention on resilience was significant (*β* = 1.346, *SE* = 0.156, *t* = 8.609, *p* < 0.01), indicating that the intervention was significantly associated with resilience.

**Table 4:** Mediation model (partially standardized).

Outcome variable	Predictor	*β*	*SE*	*t*	*R²*	*F*
Resilience (Y)	X→Y(c’)	1.118	0.171	6.520	0.502	43.83
Self-efficacy (M)	X→M	0.950	0.187	5.091	0.228	25.92
Total effect (X→Y)	X→Y(c)	1.346	0.156	8.609	0.457	74.11

*β* values represent partially standardized regression coefficients.

Bootstrap mediation results (Table 5) showed that self-efficacy partially mediated the association between the intervention and resilience (partially standardized indirect effect = 0.229, 95% CI [0.063, 0.394]; direct effect = 1.118; total effect = 1.346). The indirect effect accounted for 17% of the total effect.

**Table 5:** Result of mediation analysis.

Type of effect	Effect value	Boot SE	Bootstrap 95% CI		Effectiveness ratio	*p*
			Lower	Upper		
Direct effect	1.118	0.171	0.777	1.458	83.0%	**<0.01**
Indirect effect	0.229	0.082	0.063	0.394	17.0%	**<0.01**
Total effect	1.346	0.156	1.035	1.657	100%	**<0.01**

Effect values are partially standardized regression coefficients (Hayes, 2022). The indirect effect (0.229) represents the change in resilience (in standard deviation units) associated with the intervention via self-efficacy. Bootstrap confidence intervals (5,000 resamples) are reported. Effectiveness ratio refers to the proportion of the total effect accounted for by the indirect effect (indirect/total = 17.0%). The direct effect (83.0%) remains statistically significant after accounting for the mediator, indicating partial mediation. Bold values indicate statistically significant effects (*p* < 0.05).

## 4 Discussion

The present study found that an 8-week freestyle rope-skipping intervention was associated with significant improvements in both resilience and self-efficacy among junior high school students. At post-intervention, the experimental group exhibited significantly higher resilience and self-efficacy scores compared to the control group. Furthermore, path analysis indicated that self-efficacy served as a partial mediator in the association between the intervention and resilience. These findings suggest that freestyle rope skipping may contribute to resilience development, with improvements in self-efficacy representing one plausible explanatory pathway.

### 4.1 The impact of freestyle rope skipping intervention on the resilience of junior high school students

Freestyle rope skipping is a structured exercise intervention that integrates aerobic activity, motor coordination, rhythmic control, and progressive skill challenges. Its emphasis on sustained practice, increasing task difficulty, and flexible participation formats (individual, paired, and group-based) provides adolescents with repeated exposure to manageable stressors, opportunities for mastery, and social interaction. These features make it particularly suitable for promoting psychological resilience during early adolescence.

The results of this investigation are consistent with prior research examining similar interventions. Lin et al. (2023) reported that rope skipping significantly improved physical fitness, cardiovascular function, and exercise tolerance among adolescents with moderate intellectual disabilities, indicating its broad applicability and training effectiveness (Lin et al., 2023). Similarly, Wang et al. (2025) found that structured rope skipping interventions led to significant improvements in psychological outcomes, including emotional regulation and stress resilience, among middle school students(Wang et al., 2025). Students in the rope-skipping class showed significantly greater gains than those in the running class. Although the control group showed a slight, non-significant increase—potentially attributable to maturation or routine physical activity—the magnitude of improvement in the experimental group suggests a specific intervention effect rather than natural developmental change.

Physical exercise, as an active lifestyle behavior, can significantly improve adolescents’ physical and mental health, thereby facilitating the development of resilience (Salmon, 2001). Several interrelated mechanisms may account for these observed effects. First, the progressive challenge structure of rope skipping may expose students to manageable difficulties, allowing them to develop coping strategies through repeated practice and skill improvement (Mansuroğlu, 2025). Second, mastering increasingly complex rope-skipping skills may provide repeated mastery experiences, which are known to support confidence and psychological adaptation (Peng et al., 2025). Third, paired and group activities may enhance peer interaction and perceived social support—important contextual factors associated with resilience development.

These findings suggest that incorporating engaging physical activities such as freestyle rope skipping into school-based physical education may help support adolescents’ psychological development.

### 4.2 The impact of freestyle rope skipping intervention on the self-efficacy of junior high school students

Self-efficacy refers to an individual’s belief in their ability to successfully perform tasks and cope with challenges. Previous research has highlighted the close association between physical exercise and self-efficacy. Tikac et al. (2022) found that regular physical activity significantly improves young people’s self-efficacy, self-esteem, and body awareness. Zhang et al. (2024) further reported positive correlations among adolescent physical activity, self-efficacy, stress self-management, and mental health. In addition, Medrano-Ureña et al. (2020) noted that self-efficacy not only predicts exercise participation but also influences exercise outcomes and subsequent development. Meanwhile, Higgins et al. (2014) suggested that enhanced self-efficacy may promote sustained engagement in physical activity and help alleviate academic stress.

Building on this evidence, the present findings indicate that the 8-week freestyle rope-skipping intervention was associated with improvements in self-efficacy among junior high school students. These results are broadly consistent with previous research suggesting that structured physical activities can support self-efficacy development. Several factors may help explain this association, including opportunities for mastery experiences through progressive skill learning, peer modeling during group activities, supportive feedback from instructors, and positive emotional responses generated by regular physical activity (Chan et al., 2023; White et al., 2024; Zhang et al., 2023).

Collectively, these mechanisms help explain why freestyle rope skipping effectively enhances adolescents’ self-efficacy. Consistent with the broader exercise literature (Deng et al., 2024), the present findings suggest that incorporating engaging and structured aerobic activities such as freestyle rope skipping into school-based physical education may be an effective strategy for fostering self-efficacy and supporting adolescents’ resilience.

### 4.3 Partial mediation of self-efficacy in the freestyle rope skipping-resilience link

Previous studies have shown that self-efficacy positively contributes to resilience development among junior high school students, and that physical exercise can enhance self-efficacy, thereby indirectly promoting resilience (Qiu et al., 2025). Consistent with this evidence, the present study identified a significant positive correlation between self-efficacy and resilience. The path analysis revealed a statistical indirect effect, wherein self-efficacy explained a portion of the intervention’s association with resilience. This suggests self-efficacy may represent one behavioral pathway; however, causal inference is constrained by the observational nature of the mediator measurement. This finding extends existing research on physical exercise and psychological resilience, supporting the use of school-based exercise interventions to promote adolescent mental health.

Hu and Liu (2025) demonstrated that physical exercise promotes mental health among college students through the sequential mediating roles of self-efficacy and emotion regulation. Although their study focused on a college population using a cross-sectional design, the present findings suggest that self-efficacy may represent a psychological pathway through which physical exercise benefits mental health across different developmental stages. Similarly, An et al. (2024) argued that physical exercise not only directly improves psychological well-being but also exerts indirect effects through self-efficacy.

The observed mediating function of self-efficacy can be understood through its impact on how students appraise and respond to difficulties: it bolsters their confidence in managing challenges, sustains their effort when confronting setbacks, and shapes their tendency to interpret stressors as surmountable rather than overwhelming (Cabrera-Aguilar et al., 2023). Through repeated mastery experiences provided by freestyle rope skipping, students’ self-efficacy may be strengthened, thereby enhancing their capacity for resilient adaptation. Practical implications and interpretive caution. Although the indirect effect of self-efficacy was statistically significant, it accounted for only 17% of the total effect, leaving 83% of the intervention’s association with resilience unexplained. This modest effect size suggests that self-efficacy is one of several potential pathways, rather than a dominant explanatory mechanism. Other unmeasured variables—such as emotional regulation, social support, executive function, or physiological changes (e.g., cardiovascular fitness, neuroendocrine responses)—may also contribute to the observed improvements in resilience. From an educational practice perspective, while fostering self-efficacy is valuable, school-based interventions should not rely exclusively on this pathway. A multi-component approach targeting self-efficacy alongside other psychological and social factors may yield more robust resilience gains.

### 4.4 Limitations

Several limitations should be noted. First, the sample was drawn from two classes at one school, limiting generalizability. Second, randomization was at the class level but analyses were at the individual level without accounting for clustering, as the number of clusters (two) was insufficient for multilevel modeling. Thirdly, it was not possible to implement a double-blind design, meaning that expectancy effects may influence the outcomes. Fourthly, exercise intensity was tracked in only a subset of participants instead of being monitored continuously across all individuals. Fifth, the control group engaged in regular running as an active comparator rather than a no-intervention or passive control. This design choice was made to control for non-specific effects (e.g., time, attention, general physical activity). However, it limits our ability to draw conclusions about the absolute effect of freestyle rope skipping compared to no physical activity. The observed effects reflect the superiority of rope skipping over routine running, but do not establish whether rope skipping itself is effective relative to a true baseline. Future studies should include a no-exercise control group to address this question. Lastly, the mediator was assessed through observational methods, which means that the indirect effect illustrates a statistical correlation rather than establishing a causal relationship. Future research should focus on resolving these limitations by employing larger and more diverse samples, cluster-adjusted methodologies, passive control groups, continuous monitoring of exercise intensity, and longitudinal follow-up assessments.

## 5 Conclusion

In conclusion, participation in an 8-week freestyle rope-skipping program was associated with significant improvements in both psychological resilience and self-efficacy among junior high school students compared to those in a regular running class. Furthermore, self-efficacy emerged as a partial mediator in the intervention-resilience association, suggesting that fostering students’ efficacy beliefs constitutes one potential pathway through which school-based physical activity may enhance resilience. However, the modest indirect effect (17%) indicates that other mechanisms remain to be identified. These findings highlight the practical value of implementing enjoyable, skill-progressive rope-skipping programs in school settings, while also underscoring the need for future research with larger, more diverse samples, cluster-adjusted methodologies, and longitudinal follow-up assessments to strengthen causal inferences and examine the sustainability of these effects over time.

## Data Availability

The original contributions presented in the study are included in the article/supplementary material, further inquiries can be directed to the corresponding author.

## References

[ref1] Aguirre-Urreta M. I. Hu J. (2019). Detecting common method bias: performance of the harman’s single-factor test. SIGMIS Database 50, 45–70. doi: 10.1145/3330472.3330477

[ref3] Anderson K. Priebe S. (2021). Concepts of resilience in adolescent mental health research. J. Adolesc. Health 69, 689–695. doi: 10.1016/j.jadohealth.2021.03.035, 34045094

[ref2] An D. Pan J. Ran F. Bai D. Zhang J. (2024). Effects of physical exercise input on the exercise adherence of college students: the chain mediating role of sports emotional intelligence and exercise self-efficacy. J. Intelligence 12:94. doi: 10.3390/jintelligence12100094, 39452511 PMC11508919

[ref5] Bandura A. (1997). Self-efficacy: The exercise of control. New York, NY: Freeman.

[ref4] Bandura A. (2004). Health promotion by social cognitive means. Health Educ. Behav. 31, 143–164. doi: 10.1177/1090198104263660, 15090118

[ref6] Bonanno G. A. (2004). Loss, trauma, and human resilience: have we underestimated the human capacity to thrive after extremely aversive events? Am. Psychol. 59, 20–28. doi: 10.1037/0003-066X.59.1.20, 14736317

[ref7] Cabrera-Aguilar E. Zevallos-Francia M. Morales-García M. Ramírez-Coronel A. A. Morales-García S. B. Sairitupa-Sanchez L. Z. . (2023). Resilience and stress as predictors of work engagement: the mediating role of self-efficacy in nurses. Front. Psych. 14:1202048. doi: 10.3389/fpsyt.2023.1202048, 37649562 PMC10464840

[ref8] Chan S. Maneewan S. Koul R. (2023). An examination of the relationship between the perceived instructional behaviours of teacher educators and pre-service teachers’ learning motivation and teaching self-efficacy. Educ. Rev. 75, 264–286. doi: 10.1080/00131911.2021.1916440

[ref10] Connor K. M. Davidson J. R. T. (2003). Development of a new resilience scale: the connor-Davidson resilience scale (CD-RISC). Depress. Anxiety 18, 76–82. doi: 10.1002/da.10113, 12964174

[ref11] Costigan S. A. Eather N. Plotnikoff R. C. Hillman C. H. Lubans D. R. (2016). High-intensity interval training for cognitive and mental health in adolescents. Med. Sci. Sports Exerc. 48, 1985–1993. doi: 10.1249/MSS.0000000000000993, 27187097

[ref12] Deng L. Wu H. Ruan H. Xu D. Pang S. Shi M. (2024). Effects of fancy rope-skipping on motor coordination and selective attention in children aged 7–9 years: a quasi-experimental study. Front. Psychol. 15:1383397. doi: 10.3389/fpsyg.2024.1383397, 39171233 PMC11337131

[ref13] Deuster P. A. Silverman M. N. (2013). Physical fitness: a pathway to health and resilience. U. S. Army Med. Dep. J., 24–35. 24146240

[ref14] Epstein R. M. Krasner M. S. (2013). Physician resilience: what it means, why it matters, and how to promote it. Acad. Med. J. Assoc. Am. Med. Coll. 88, 301–303. doi: 10.1097/ACM.0b013e318280cff0, 23442430

[ref15] Fenwick-Smith A. Dahlberg E. E. Thompson S. C. (2018). Systematic review of resilience-enhancing, universal, primary school-based mental health promotion programs. BMC Psychol. 6:30. doi: 10.1186/s40359-018-0242-3, 29976252 PMC6034212

[ref9002] Hayes A. F. (2022). Introduction to mediation, moderation, and conditional process analysis: A regression-based approach (2nd ed.). New York, NY: Guilford Press.

[ref9001] Hayes A. F. (2013). Introduction to mediation, moderation, and conditional process analysis: A regression-based approach. New York, NY: Guilford Press.

[ref16] Higgins T. J. Middleton K. R. Winner L. Janelle C. M. (2014). Physical activity interventions differentially affect exercise task and barrier self-efficacy: a meta-analysis. Health Psychol. 33, 891–903. doi: 10.1037/a0033864, 23957904 PMC4148031

[ref18] Huang Z. Li L. Lu Y. Meng J. Wu X. (2024). Effects of rope skipping exercise on working memory and cardiorespiratory fitness in children with attention deficit hyperactivity disorder. Front. Psych. 15:1381403. doi: 10.3389/fpsyt.2024.1381403, 38846914 PMC11153777

[ref17] Hu Y. Liu B. (2025). The impact of physical exercise on college students’ mental health through emotion regulation and self-efficacy. Sci. Rep. 15:33548. doi: 10.1038/s41598-025-18352-9, 41022943 PMC12480569

[ref19] Jiang C. Wang K. Qin H. (2025). Physical exercise and children’s resilience: mediating roles of self-efficacy and emotional intelligence. Front. Psychol. 16, 1–10. doi: 10.3389/fpsyg.2025.1491262, 40191570 PMC11968682

[ref20] Kaniasty K. Benight C. C. van der Meulen E. (2025). Future coping self-efficacy as proxy for resilience. Appl. Psychol. Health Well-Being 17:e70028. doi: 10.1111/aphw.70028, 40202164

[ref21] Kieling C. Buchweitz C. Caye A. Silvani J. Ameis S. H. Brunoni A. R. . (2024). Worldwide prevalence and disability from mental disorders across childhood and adolescence. JAMA Psychiatr. 81, 347–356. doi: 10.1001/jamapsychiatry.2023.5051, 38294785 PMC10831630

[ref22] Lee M. Evans M. (2019). Investigating the operating mechanisms of the sources of L2 writing self-efficacy at the stages of giving and receiving peer feedback. Mod. Lang. J. 103, 831–847. doi: 10.1111/modl.12598

[ref23] Li N. Wang D. Zhao X. Li Z. Zhang L. (2024). The association between physical exercise behavior and psychological resilience of teenagers: An examination of the chain mediating effect. Sci. Rep. 14:9372. doi: 10.1038/s41598-024-60038-1, 38654069 PMC11039466

[ref24] Lin Y.-Y. Su C.-T. Liao Y.-H. Liu Y.-C. (2023). Effects of rope skipping exercise on physical, cardiovascular fitness and exercise tolerance in adolescent students with moderate intellectual disability. J. Intellect. Disabil. Res. JIDR 67, 1136–1149. doi: 10.1111/jir.13071 37578101

[ref25] Mansuroğlu S. (2025). The effectiveness of stress management training given to first-class health major students in perceiving and coping with stress and developing resilience: a randomized controlled trial. Appl. Psychol. Health Well-Being 17:e70014. doi: 10.1111/aphw.70014, 40042083 PMC11881215

[ref26] Marconcin P. Werneck A. O. Peralta M. Ihle A. Gouveia É. R. Ferrari G. . (2022). The association between physical activity and mental health during the first year of the COVID-19 pandemic: a systematic review. BMC Public Health 22:209. doi: 10.1186/s12889-022-12590-6, 35101022 PMC8803575

[ref27] Maria S. A. Nicolae O. M. Nicola M. Szekely A. S. Sorin S. Dorina I. . (2025). Jump rope training improves muscular strength and cardiovascular fitness in university students: a controlled educational intervention. Sports 13:307. doi: 10.3390/sports13090307, 41003613 PMC12473967

[ref28] Medrano-Ureña M. d. R. Ortega-Ruiz R. Benítez-Sillero J. d. D. (2020). Physical fitness, exercise self-efficacy, and quality of life in adulthood: a systematic review. Int. J. Environ. Res. Public Health 17:6343. doi: 10.3390/ijerph17176343, 32878182 PMC7504332

[ref29] Nobre J. Oliveira A. P. Monteiro F. Sequeira C. Ferré-Grau C. (2021). Promotion of mental health literacy in adolescents: a scoping review. Int. J. Environ. Res. Public Health 18:9500. doi: 10.3390/ijerph18189500, 34574427 PMC8470967

[ref30] Panter-Brick C. Leckman J. F. (2013). Editorial commentary: resilience in child development--interconnected pathways to wellbeing. J. Child Psychol. Psychiatry 54, 333–336. doi: 10.1111/jcpp.12057, 23517424

[ref31] Peng B. Chen W. Wang H. Yu T. (2025). How does physical exercise influence self-efficacy in adolescents? A study based on the mediating role of psychological resilience. BMC Psychol. 13, 285–301. doi: 10.1186/s40359-025-02529-y, 40119462 PMC11927186

[ref33] Pujari V. (2024). Moving to improve mental health - the role of exercise in cognitive function: a narrative review. J. Pharm. Bioallied Sci. 16, S26–S30. doi: 10.4103/jpbs.jpbs_614_23, 38595617 PMC11000952

[ref34] Qiu W. Wang X. Cui H. Ma W. Xiao H. Qu G. . (2025). The impact of physical exercise on college students’ physical self-efficacy: the mediating role of psychological resilience. Behav. Sci. 15:541. doi: 10.3390/bs15040541, 40282162 PMC12024398

[ref35] Rodgers W. M. Markland D. Selzler A.-M. Murray T. C. Wilson P. M. (2014). Distinguishing perceived competence and self-efficacy: an example from exercise. Res. Q. Exerc. Sport 85, 527–539. doi: 10.1080/02701367.2014.961050, 25412135

[ref36] Ruby M. B. Dunn E. W. Perrino A. Gillis R. Viel S. (2011). The invisible benefits of exercise. Health Psychol. 30, 67–74. doi: 10.1037/a0021859, 21299296

[ref37] Sagone E. De Caroli M. E. Falanga R. Indiana M. L. (2020). Resilience and perceived self-efficacy in life skills from early to late adolescence. Int. J. Adolesc. Youth 25, 882–890. doi: 10.1080/02673843.2020.1771599

[ref38] Salmon P. (2001). Effects of physical exercise on anxiety, depression, and sensitivity to stress: a unifying theory. Clin. Psychol. Rev. 21, 33–61. doi: 10.1016/S0272-7358(99)00032-X, 11148895

[ref39] Santos R. M. S. Mendes C. G. Sen Bressani G. Y. de Alcantara Ventura S. de Almeida Nogueira Y. J. de Miranda D. M. . (2023). The associations between screen time and mental health in adolescents: a systematic review. BMC Psychol. 11:127. doi: 10.1186/s40359-023-01166-7, 37081557 PMC10117262

[ref40] Serey J. Morand G. B. Danuser B. (2019). The relationship between physical activity and resilience: A systematic review. Frontiers in Psychology, 10:2562. doi: 10.3389/fpsyg.2019.02562 31803107 PMC6874039

[ref41] Steare T. O’Connor D. B. Ferguson E. (2023). Physical activity, mental health, and resilience: A longitudinal study. Journal of Affective Disorders, 323, 298–305. doi: 10.1016/j.jad.2022.11.047

[ref42] Tikac G. Unal A. Altug F. (2022). Regular exercise improves the levels of self-efficacy, self-esteem and body awareness of young adults. J. Sports Med. Phys. Fitness 62, 157–161. doi: 10.23736/S0022-4707.21.12143-7, 33555673

[ref43] Wang K. Li Y. Zhang T. Luo J. (2022). The relationship among college students’ physical exercise, self-efficacy, emotional intelligence, and subjective well-being. Int. J. Environ. Res. Public Health 19:11596. doi: 10.3390/ijerph191811596, 36141869 PMC9517190

[ref44] Wang T. Nie Y. Yao X. Zhang J. Li Y. Sun H. . (2025). The chain mediating role of emotion regulation and stress perception in physical activity alleviating college students’ health anxiety. Sci. Rep. 15:29189. doi: 10.1038/s41598-025-14481-3, 40783609 PMC12335499

[ref45] White R. L. Vella S. Biddle S. Sutcliffe J. Guagliano J. M. Uddin R. . (2024). Physical activity and mental health: a systematic review and best-evidence synthesis of mediation and moderation studies. Int. J. Behav. Nutr. Phys. Act. 21:134. doi: 10.1186/s12966-024-01676-6, 39609855 PMC11603721

[ref9003] Xiao S. Y. Zhang M. Y. (2007). Reliability and validity of the Chinese version of the Connor-Davidson Resilience Scale (CD-RISC). Chinese Journal of Clinical Psychology, 15, 234–236.

[ref46] Xia Q. Liu Q. Qin G. (2024). The mediating role of psychological resilience in the relationship between physical exercise and sense of security among left-behind junior high school students: multi-group comparative analysis of only children and children with siblings. Front. Psychol. 15:1411175. doi: 10.3389/fpsyg.2024.1411175, 39698385 PMC11654250

[ref9004] Xia Y. Zhang S. Wu Q. (2019). Prevalence of mental disorders in children and adolescents in China: Diagnostic data from detailed clinical assessments of 17,524 individuals. The Lancet Psychiatry, 6, 810–817. doi: 10.1016/S2215-0366(19)30206-1 34019305

[ref47] Yubin Y. Omar Dev R. D. Geok S. K. XueYan J. (2025). Enhancing exercise adherence through self-efficacy: mechanisms, moderators, and interventions. Int. J. Acad. Res. Bus. Soc. Sci. 15, 1713–1722. doi: 10.6007/IJARBSS/v15-i3/25122

[ref48] Zhang G. Feng W. Zhao L. Zhao X. Li T. (2024). The association between physical activity, self-efficacy, stress self-management and mental health among adolescents. Sci. Rep. 14:5488. doi: 10.1038/s41598-024-56149-4, 38448518 PMC10917799

[ref9005] Zhang Y. Wang J. Liu H. Chen X. (2023). Cognitive enhancement through differential rope skipping after math lesson. International Journal of Environmental Research and Public Health, 20: 205. doi: 10.3390/ijerph20010205 PMC9819879 36612527

[ref49] Zhu F. Zhu X. Bi X. Kuang D. Liu B. Zhou J. . (2023). Comparative effectiveness of various physical exercise interventions on executive functions and related symptoms in children and adolescents with attention deficit hyperactivity disorder: a systematic review and network meta-analysis. Front. Public Health 11:1133727. doi: 10.3389/fpubh.2023.1133727, 37033046 PMC10080114

